# Shared genetic susceptibility between trigger finger and carpal tunnel syndrome: a genome-wide association study

**DOI:** 10.1016/S2665-9913(22)00180-1

**Published:** 2022-08

**Authors:** Benjamin Patel, Sam O Kleeman, Drew Neavin, Joseph Powell, Georgios Baskozos, Michael Ng, Waheed-Ul-Rahman Ahmed, David L Bennett, Annina B Schmid, Dominic Furniss, Akira Wiberg

**Affiliations:** Department of Plastic and Reconstructive Surgery, Southmead Hospital, North Bristol NHS Trust, Bristol, UK; Cold Spring Harbor Laboratory, New York, NY, USA; Garvan-Weizmann Centre for Cellular Genomics, Garvan Institute of Medical Research, Sydney, NSW, Australia; Garvan-Weizmann Centre for Cellular Genomics, Garvan Institute of Medical Research, Sydney, NSW, Australia; UNSW Cellular Genomics Futures Institute, University of New South Wales, NSW, Australia; Nuffield Department of Clinical Neurosciences, John Radcliffe Hospital, University of Oxford, Oxford, UK; Nuffield Department of Orthopaedics, Rheumatology and Musculoskeletal Sciences, Botnar Research Centre, University of Oxford, Oxford, UK; Nuffield Department of Orthopaedics, Rheumatology and Musculoskeletal Sciences, Botnar Research Centre, University of Oxford, Oxford, UK; Nuffield Department of Clinical Neurosciences, John Radcliffe Hospital, University of Oxford, Oxford, UK; Nuffield Department of Clinical Neurosciences, John Radcliffe Hospital, University of Oxford, Oxford, UK; Nuffield Department of Orthopaedics, Rheumatology and Musculoskeletal Sciences, Botnar Research Centre, University of Oxford, Oxford, UK; Nuffield Department of Orthopaedics, Rheumatology and Musculoskeletal Sciences, Botnar Research Centre, University of Oxford, Oxford, UK

## Abstract

**Background:**

Trigger finger and carpal tunnel syndrome are the two most common non-traumatic connective tissue disorders of the hand. Both of these conditions frequently co-occur, often in patients with rheumatoid arthritis. However, this phenotypic association is poorly understood. Hypothesising that the co-occurrence of trigger finger and carpal tunnel syndrome might be explained by shared germline predisposition, we aimed to identify a specific genetic locus associated with both diseases.

**Methods:**

In this genome-wide association study (GWAS), we identified 2908 patients with trigger finger and 436579 controls from the UK Biobank prospective cohort. We conducted a case-control GWAS for trigger finger, followed by co-localisation analyses with carpal tunnel syndrome summary statistics. To identify putative causal variants and establish their biological relevance, we did fine-mapping analyses and expression quantitative trait loci (eQTL) analyses, using fibroblasts from healthy donors (n=79) and tenosynovium samples from patients with carpal tunnel syndrome (n=77). We conducted a Cox regression for time to trigger finger and carpal tunnel syndrome diagnosis against plasma IGF-1 concentrations in the UK Biobank cohort.

**Findings:**

Phenome-wide analyses confirmed a marked association between carpal tunnel syndrome and trigger finger in the participants from UK Biobank (odds ratio [OR] 11·97, 95% CI 11·1–13·0; p<1 × 10^–300^). GWAS for trigger finger identified five independent loci, including one locus, *DIRC3*, that was co-localised with carpal tunnel syndrome and could be fine-mapped to rs62175241 (0·76, 0·68–0·84; p=5·03 × 10^–13^). eQTL analyses found a fibroblast-specific association between the protective T allele of rs62175241 and increased *DIRC3* and *IGFBP5* expression. Increased plasma IGF-1 concentrations were associated with both carpal tunnel syndrome and trigger finger in participants from UK Biobank (hazard ratio >1·04, p<0·02).

**Interpretation:**

In this GWAS, the *DIRC3* locus on chromosome 2 was significantly associated with both carpal tunnel syndrome and trigger finger, possibly explaining their co-occurrence. The disease-protective allele of rs62175241 was associated with increased expression of long non-coding RNA *DIRC3* and its transcriptional target, *IGBP5*, an antagonist of IGF-1 signalling. These findings suggest a model in which IGF-1 is a driver of both carpal tunnel syndrome and trigger finger, and in which the *DIRC3-IGFBP5* axis directly antagonises fibroblastic IGF-1 signalling.

**Funding:**

Wellcome Trust, National Institute for Health Research, Medical Research Council.

## Introduction

Trigger finger (also known as stenosing flexor tenosynovitis) and carpal tunnel syndrome are the two most common non-traumatic hand disorders, with a lifetime prevalence of 2–10%^1^ for trigger finger and 3–10% for carpal tunnel syndrome.^2^ Trigger finger is caused by impaired gliding of the flexor tendons through the first annular (A1) pulley and manifests as painful clicking of the digit during flexion and extension, with progressive stiffness, locking, and loss of function. Carpal tunnel syndrome is a compression neuropathy of the median nerve that manifests as paresthesia, pain, numbness, and weakness of the hand. Both conditions are associated with marked functional impairment and reduced quality of life. Carpal tunnel syndrome is associated with an estimated loss of 78 375 disease-adjusted life years annually in the USA, and a total disease burden of US$2·7 to $4·8 billion per year.^3,4^

Observational studies have provided evidence to suggest that there is a link between carpal tunnel syndrome and trigger finger: a prospective study found that 43% of patients diagnosed with trigger finger had clinical signs or symptoms of carpal tunnel syndrome,^4^ and another study of patients with carpal tunnel syndrome or trigger finger found that, on clinical examination, 61% of patients had both carpal tunnel syndrome and trigger finger.^5^ 63% of patients with trigger finger in another study had neurophysiological evidence of carpal tunnel syndrome compared with only 8% of controls.^6^ Although some studies have argued that the risk of trigger finger is increased after carpal tunnel syndrome surgical decompression, which could lead to confounding of the observed association, a retrospective longitudinal study from 2019 showed no temporal correlation between ipsilateral trigger finger and carpal tunnel syndrome surgery.^7^ Both conditions are characterised by synovitis and fibrosis around the flexor tendons^8^ and share multiple risk factors, including repetitive movements, diabetes, obesity, pregnancy, and rheumatoid arthritis.^1,9^ Furthermore, trigger finger and carpal tunnel syndrome can both be treated with a corticosteroid injection and surgical decompression.

The association between carpal tunnel syndrome and trigger finger is incompletely characterised. We hypothesised that the co-occurrence of these conditions might be explained by a common genetic risk locus. Although the genetic architecture of carpal tunnel syndrome has been investigated through a genome-wide association study (GWAS) using data from UK Biobank,^10^ much less is known about the genetic basis of trigger finger. The only published GWAS for trigger finger included 942 patients and 24 472 controls, and identified a single non-replicated locus on chromosome 13.^11^ In this GWAS, we aimed to leverage the UK Biobank cohort to identify a specific genetic locus associated with both conditions that could potentially explain their co-occurrence.

## Methods

### Study design and participants

**Figure 1:**
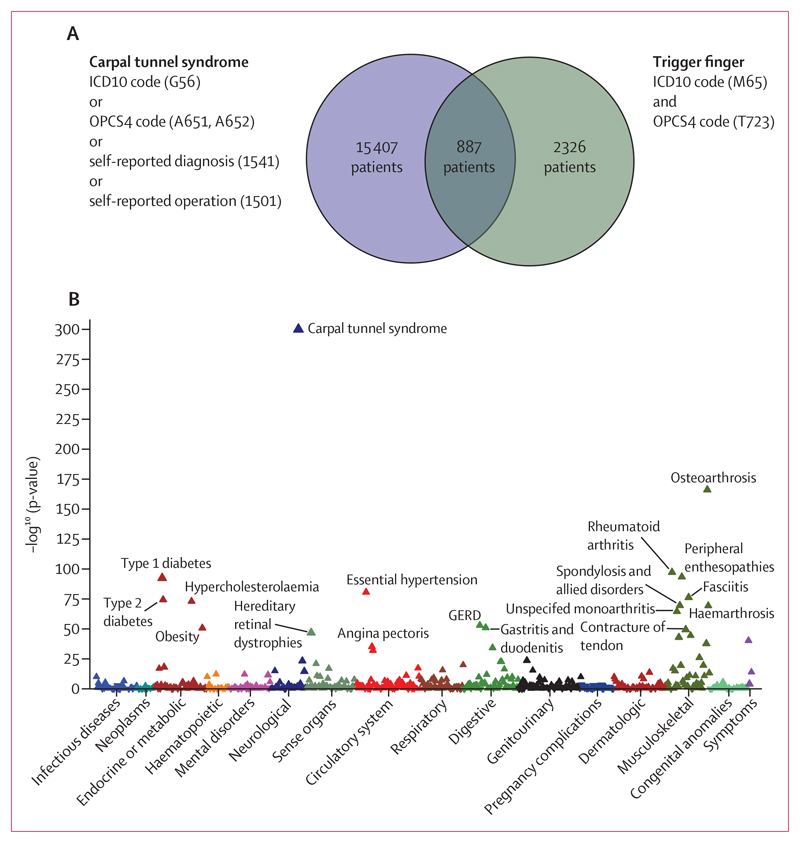
Association between trigger finger and carpal tunnel syndrome Data from UK Biobank. (A) Overlap between trigger finger and carpal tunnel syndrome in UK Biobank, annotated with the International Classification of Diseases (ICD)-10 codes and Office of Population Censuses and Surveys Classification of Surgical Operations and Procedures (OPCS) codes that were used to define whether patients had carpal tunnel syndrome or trigger finger. (B) Phenome-wide association analysis, showing the association between trigger finger and 694 phenotypes that were derived from ICD10 coding in UK Biobank. The p-value refers to a Fisher’s exact test, and the direction of the triangle reflects the direction of the effect. GERD=gastroesophageal reflux disease.

Patients with carpal tunnel syndrome (n=16 294) were defined as previously described.^10^ To maximise specificity, patients with trigger finger were defined by the intersection of patients who had International Classification of Diseases(ICD)-10 codes for trigger finger (M65.3, M65.30-39), and patients who had Office of Population Censuses and Surveys Classification of Surgical Operations and Procedures (OPCS) codes for tendon release (T723; figure 1A). In sensitivity analyses, we explored including patients who had either ICD-10 or OPCS codes alone (termed the extended cohort). We also included patients with self-reported trigger finger symptoms (UK Biobank self-reported illness code 1619), termed the mixed cohort. Further information for cohort definitions is provided in the appendix (p 2). To define a control group for all subsequent genome-wide analyses, we selected 488 263 participants from UK Biobank with imputed genomic data, excluding 31657 participants who did not pass genomic quality control and 20 027 participants who had coding of any nature for either carpal tunnel syndrome of trigger finger (appendix p 12).

UK Biobank has approval from the North West Multi-Centre Research Ethics Committee (11/NW/0382), and this study was conducted under UK Biobank study ID 22572. The Oxford carpal tunnel syndrome cohort was derived from two clinical studies that were approved by the National Research Ethics Service (UK): the Pain in Neuropathy Study (PiNS; 10/H07056/35), and the Molecular Genetics of Carpal Tunnel Syndrome (MGCTS) study (16/LO/1920). Written informed consent was obtained from all participants recruited to these studies. Genomic data quality control steps for the UK Biobank trigger finger cohort is summarised in the appdendix (p 12).

### Phenotypic association analysis

To identify diagnoses associated with trigger finger, all UK Biobank first occurrence fields (a specific UK Biobank data resource) and cancer registry data (fields 40 005 and 40 006) were extracted, with ICD-10 codes mapped to Phecodes.^12^ Data entries were binarised to construct a matrix of 694 diagnosis codes (including trigger finger and carpal tunnel syndrome, as defined above) versus 502 505 participants from UK Biobank. To determine the association between trigger finger diagnosis pairs, we constructed a 2 × 2 contingency table for each pair and did a Fisher’s one-way test. Significance level was set at p<1 × 10^–5^ which met the appropriate Bonferroni-adjusted threshold for the 694 phenotypes tested.^13^

### Genome-wide association analyses

Genome-wide association analyses were implemented in Regenie (version 2.2.1)^14^ in the European ancestry cohort using Firth approximation, with covariates including year of birth (field 34), genotyping array (which were binarised from field 22000), recruitment centre (field 54), and principal components one to ten. For phenome-wide association analysis using data from UK Biobank, summary statistics were exported from the OpenTargets Genetics Portal,^15^ extracting data specific to the European ancestry group and with p<1 × 10^–5^.

### Processing of summary statistics

To identify independent signals, we conducted conditional and joint analyses, implemented in Genome-wide Complex Trait Analysis(GCTA)-Conditional and Joint analysis (COJO)^16^ using a linkage disequilibrium reference derived from 1000 Genomes. Linkage disequilibrium score regression was implemented in the ldsc package for R using a UK Biobank European ancestry linkage disequilibrium reference derived from the PanUK Biobank project. Lead single-nucleotide polymorphisms (SNPs) were annotated using the OpenTargets Genetics portal, which considered three annotations: the nearest coding gene, genes with a cis-expression quantitative trait loci (eQTL) variant in linkage disequilibrium (*r*^2^>0·8) with the lead SNP, and the Variant2Gene score.

### Co-localisation analysis

Carpal tunnel syndrome summary statistics from our previous GWAS were used.^10^ To extract data that were specific to the *DIRC3* locus, we filtered the merged summary statistics to a 1MB region centered at rs10203066. To extract a signed linkage disequilibrium correlation matrix for the SNPs in these regions, we used the ld_matrix_local function in the ieugwasr package in R using a linkage disequilibrium reference derived from 5000 randomly selected unrelated European participants from UK Biobank. Co-localisation analyses were implemented in the coloc package in R using the coloc. susie function, with default parameters.^17^ The posterior probability for hypothesis 4 (H4), reflecting the existence of a shared causal variant, was extracted. To determine the 95% credible set of co-localised variants, we extracted the posterior probabilities of each SNP, conditioned on H4 being true. These SNPs were functionally annotated using ensembl variant effect predictor in the Ensembl web server.

### Replication in FinnGen cohort and meta-analysis

Summary statistics for trigger finger (M13) and carpal tunnel syndrome (G6) were downloaded from the FinnGen portal (release 4).^18^ The FinnGen analysis pipeline has been described previously.^19^ Plots were generated in LocusZoom using legacy mode and the 1000 Genomes European linkage disequilibrium reference, relative to the index SNP rs10203066. We did a sample size-weighted meta-analysis between our UK Biobank trigger finger summary statistics (core analysis) and FinnGen R4 trigger finger summary statistics, which we then implemented in METAL (version March 2011) using the analyse heterogeneity command.^20^

### Oxford-carpal tunnel syndrome cohort sample collection

Sample collection for both the PiNS and MGCTS has been described previously.^10^ Briefly, patients with clinically diagnosed carpal tunnel syndrome underwent carpal tunnel decompression surgery, during which tenosynovial specimens were collected. Samples were preserved in RNAlater (Thermo Fisher, MA, USA) before extraction using the High Pure RNA Isolation Kit (Roche, Basel, Switzerland). For paired whole-genome genotyping, DNA was extracted from whole blood samples using the PureLink Genomic DNA Kit (Invitrogen, MA, USA).

### RNA sequencing

RNA extraction and library preparation were done as described previously.^10^ Reads were aligned to the GRCh37 reference with STAR^21^ using the Ensembl 87 gene annotation, with gene-level counts assigned using HTSeq.^22^ Count-level batch correction between MGCTS and PiNS cohorts was done using ComBat-seq. To facilitate inter-sample comparisons, count-level data was Trimmed Mean of the M-values(TMM)-normalised and log-transformed to generate log-transcripts per million (log-counts per million [CPM]) data.

### eQTL analysis

Harmonised summary statistics from our analysis of Genotype-Tissue Expression (GTEx) project data were downloaded from the eQTL Catalogue.^23^ eQTL analysis for *IGFBP5* in the cohort analysed by Neavin and colleagues was done as described previously.^24^ Briefly, for each individual and fibroblast cluster, the quantile-normalised pseudobulk average expression was extracted, and cis-eQTL association statistics were computed using a linear model implemented in MatrixEQTL,^25^ with one PEER factor as covariate. eQTL analysis for *DIRC3* and *IGFBP5* in the Oxford-carpal tunnel syndrome cohort was implemented as a Kruskal-Wallis test for gene expression (log-CPM) against genotype.

### IGF-1 in UK Biobank

IGF-1 plasma levels from the first recruitment visit were extracted from field 30770 and were normalised by Z-scoring, stratified by age (decile) and sex. For Cox regression of trigger finger syndrome-free survival or carpal tunnel syndrome-free survival against Z-scored IGF-1, the time variable used was time from blood sampling to ICD-coded diagnosis of carpal tunnel syndrome or trigger finger, or last follow-up date. Last follow-up date was determined through integration of death status, recruitment visits, and ICD coding dates. The model was adjusted for age at first recruitment visit, sex, and recruitment centre.

### Role of the funding source

The funders of the study had no role in study design, data collection, data analysis, data interpretation, or writing of the report.

## Results

Phenome-wide analyses were conducted across the whole UK Biobank cohort (n=502 490), which recapitulated a highly significant association between trigger finger and carpal tunnel syndrome (p<1×10^−300^ [odds ratio (OR) 11·97], 95% CI 11·1–13·0; figure 1B). To explore the phenotypic association between trigger finger and carpal tunnel syndrome, we quantified the overlap between trigger finger and carpal tunnel syndrome (figure 1A) and explored the clinical characteristics of patients with trigger finger, carpal tunnel syndrome, and trigger finger–carpal tunnel syndrome overlap (appendix p 3). We identified evidence for a significantly increased prevalence of type 1 diabetes and type 2 diabetes, as well as significantly increased HbA1c levels (a routinely used biomarker for glycaemic control) in patients with trigger finger–carpal tunnel syndrome overlap compared with patients with carpal tunnel syndrome alone (p<1 × 10^–4^) and patients with trigger finger alone (p<0·01).

**Figure 2:**
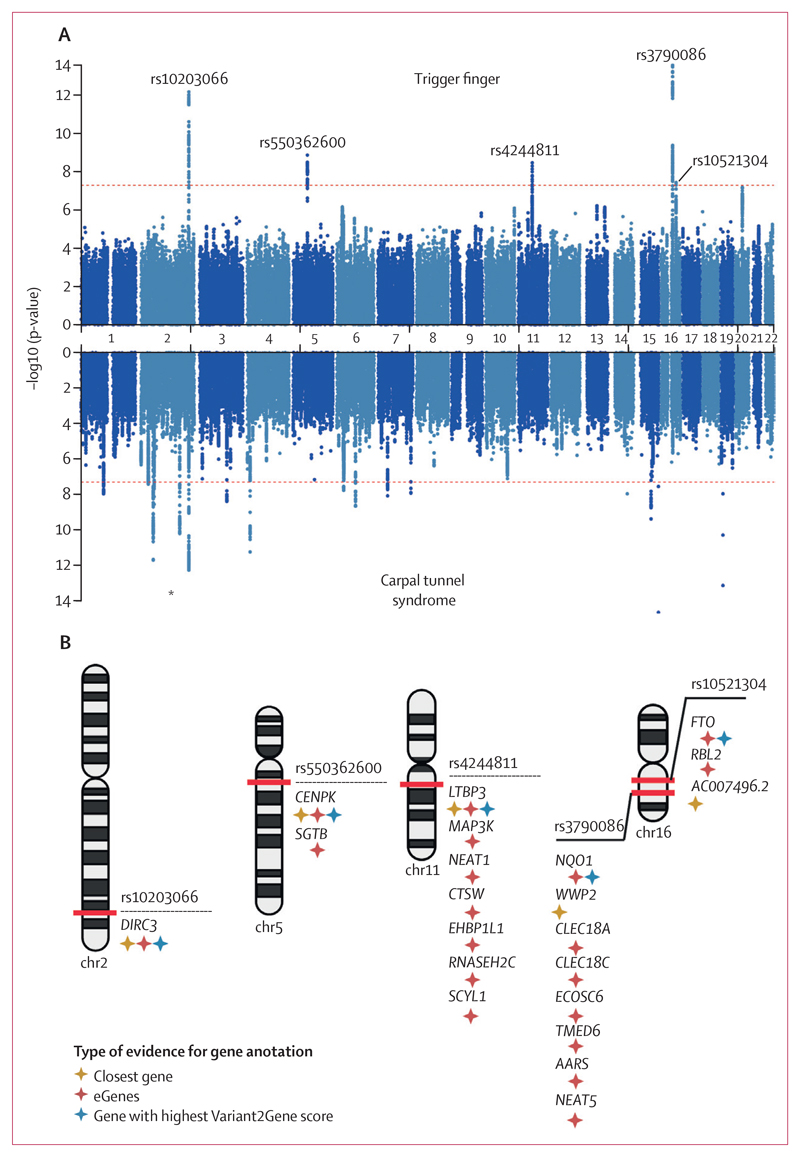
Genome-wide association results for trigger finger and carpal tunnel syndrome (A) Summary statistics from trigger finger and carpal tunnel syndrome (derived from Wiberg and colleagues) genome-wide association analyses in European patients from UK Biobank. Independent loci in trigger finger summary statistics are annotated with the single-nucleotide polymorphism (SNP) identifier for the index SNP (lowest p-value) at each locus. (B) Results of gene prioritisation analysis for trigger finger trait, using multiple annotations including nearest gene to SNP, cis-eQTL associations and the Variant2Gene score from OpenTargets Genetics. AARS=alanyl-tRNA synthetase. CENPK=centromere protein K. CLEC18A=C-Type Lectin Domain Family 18 Member A. CLEC18C=C-Type Lectin Domain Family 18 Member C. CTSW=cathepsin W. DIRC3=Disrupted In Renal Carcinoma 3. EHBP1L1=EH domain binding protein 1 like 1. FTO=FTO alpha-ketoglutarate dependent dioxygenase. LTBP3=latent transforming growth factor beta binding protein 3. MAP3K=mitogen-activated protein kinase kinase kinase 1. NEAT1=nuclear paraspeckle assembly transcript 1. NEAT5=nuclear paraspeckle assembly transcript 5. NQO1=NAD(P)H quinone dehydrogenase 1. RBL2=RB transcriptional corepressor like 2. RNASEH2C=ribonuclease H2 subunit C. SCYL1=SCY1 like pseudokinase 1. SGTB=small glutamine rich tetratricopeptide repeat co-chaperone beta. TMED6=transmembrane p24 trafficking protein 6. WWP2=WW domain containing E3 ubiquitin protein ligase 2. *Signal at *DIRC3* locus common to trigger finger and carpal tunnel syndrome analyses.

To investigate the genetic architecture of trigger finger, we conducted a GWAS in participants with European ancestry (n=456 606) from UK Biobank, incorporating 2908 patients with trigger finger (as previously defined [figure 1A]) who passed sample quality control (appendix p 12), and 436 579 controls (appendix p 2) using a mixed-model approach that accounted for unbalanced case-control ratios, population structure, and cryptic relatedness. There were insufficient numbers of patients with trigger finger in non-European ancestry groups to conduct a sufficiently powered analysis. There was also no evidence of significant confounding, with a genomic inflation factor (λ_GC_ 1·058), and we estimated the SNP-based heritability for trigger finger to be 0·8% (SE 0·1%). We identified five independent risk loci, comprising 419 variants, that met genome-wide significance (figure 2A). Conditional analyses showed no evidence of secondary signals at each locus. Using a multi-modal approach to gene-mapping, we identified 21 candidate genes at the five loci (figure 2B; appendix p 6).

In support of our stringent approach to trigger finger phenotype definition, we found that conducting the GWAS by including patients with either ICD-10 or OPCS codes (ie, the extended cohort) or additionally including patients with self-reported trigger finger (ie, the mixed cohort) markedly reduced the power to detect significant associations across the five loci (appendix p 2, 6).

**Figure 3:**
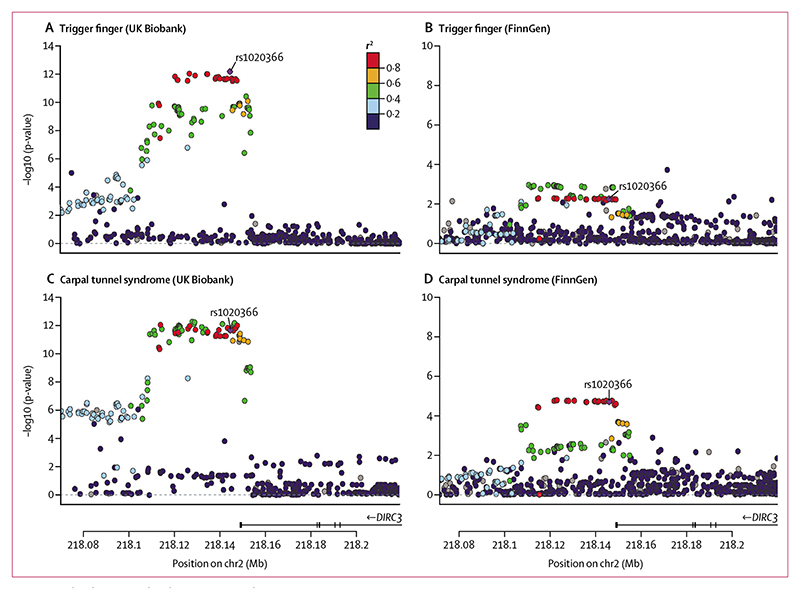
Co-localisation and replication at *DIRC3* locus Single-nucleotide polymorphism(SNP)-level associations with trigger finger (A, B) and carpal tunnel syndrome (C, D) at *DIRC3* locus, displayed as a LocusZoom plot, derived from UK Biobank (A, C) and FinnGen (B, D) prospectively-recruited cohorts. Index SNP at *DIRC3* locus (rs10203066) is annotated in purple, and each SNP is coloured according to *r*^2^ with rs10203066, derived from the 1000 Genomes linkage disequilibrium reference. DIRC3=Disrupted In Renal Carcinoma 3. Mb=megabase.

At the *DIRC3* locus (encoding a long non-coding RNA [lncRNA]), index SNP rs10203066 (p=6·73 × 10^–13^ [OR 0·75]; 95% CI 0·69–0·82) was shared with our GWAS on carpal tunnel syndrome (p=2·20 × 10^–12^ [0·88]; 0·85–0·91; figure 3A, C).^10^ To confirm that this signal was not driven solely by patients with carpal tunnel syndrome in our trigger finger cohort, we undertook a further sensitivity GWAS analysis excluding any patients with carpal tunnel syndrome (appendix p 2) and confirmed that this signal retained genome-wide significance (p=1·09 × 10^–10^; appendix p 6). Consistent with the independent association of this locus with carpal tunnel syndrome and trigger finger, we confirmed an increase in statistical significance by merging all patients with trigger finger and carpal tunnel syndrome (p=1·28 × 10^–24^; appendix p 6).

We conducted a genetic correlation analysis using linkage disequilibrium score regression and identified evidence of a high correlation between trigger finger and carpal tunnel syndrome traits (genetic correlation estimate 0·70). To identify the loci that were driving this genetic correlation, we conducted a co-localisation analysis with a multiple causal variant assumption (SuSiE coloc). This analysis identified a high (87%) posterior probability that trigger finger and carpal tunnel syndrome share a single causal variant at this locus. To replicate the association between the *DIRC3* locus and both trigger finger and carpal tunnel syndrome, we extracted summary statistics for trigger finger and carpal tunnel syndrome from the FinnGen cohort (release 4; https://finngen.gitbook.io/documentation/v/r4/). The association with both trigger finger (1485 patients and 137185 controls; p=0·0055 [OR 0·88]; 95% CI 0·81–0·96; appendix p 6) and carpal tunnel syndrome (8576 patients and 158705 controls; p=1·90 × 10^–5^ [0·91]; 0·88–0·95) was confirmed using a Bonferroni-corrected threshold of p<0·01 (figure 3B, D). A formal meta-analysis of UK Biobank and FinnGen summary statistics for trigger finger showed directional concordance at all loci, with low heterogeneity across three out of five loci (appendix p 7).

We leveraged the co-localisation analysis between the traits for carpal tunnel syndrome and trigger finger to fine-map the *DIRC3* locus by extracting the 95% credible set (n=21) of co-localised variants (appendix pp 8–9). Next, using Ensembl Variant Effector Predictor,^26^ we functionally annotated the SNPs with their immediate regulatory environment. One SNP, rs62175241, had significant regulatory consequences by disrupting an enhancer site active in fibroblasts, as well as the binding motifs for a range of transcription factors, including *KLF16* and *KLF18* (appendix p 10).

**Figure 4:**
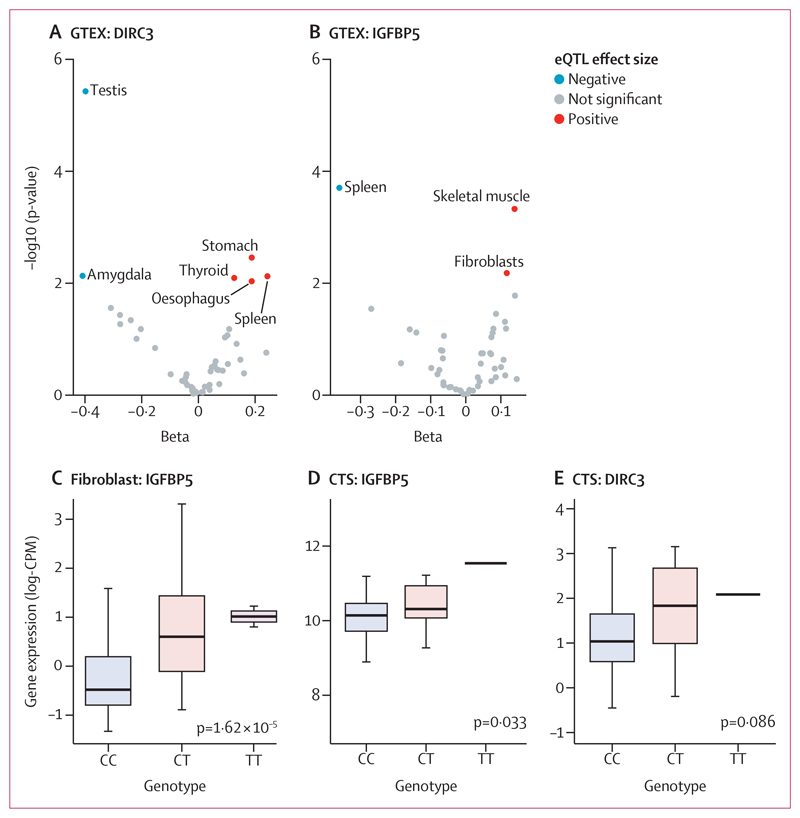
rs62175241 is associated with increased RNA expression the *DIRC3-IGFBP5* axis Cis-eQTL analysis for rs62175241 on *DIRC3* (A) and *IGFBP5* (B) gene expression in 53 tissues from the GTEx project. Tissue-specific eQTL associations with p values <0·01 are annotated (ie, we have labelled the tissue type for significant associations). p values refer to the linear model, adjusted for PC1-6. (C) Validation of rs62175241 as an eQTL for *IGFPB5* expression in the *HOXC6*+ fibroblast cluster, using single-cell RNA sequencing of fibroblasts from 79 donors. The p value refers to the linear model, adjusted for average expression and 1 PEER factor. Association between rs62175241 genotype and *DIRC3* (D) and *IGFBP5* (E) expression, derived from paired whole-genome genotyping and RNA sequencing of surgical tenosynovium samples in the Oxford-carpal tunnel syndrome cohort (n=77, including 18 patients with CT genotype and one patient with TT genotype). The p value refers to Kruskal-Wallis test. CPM=counts per million. CTS=carpal tunnel syndrome. DIRC3=Disrupted In Renal Carcinoma 3. eQTL=expression quantitative trait. GTEX=genotype-tissue expression. IGFBP5=Insulin-like growth factor-binding protein-5.

To investigate how rs62175241 (p=5·03 × 10^–13^ [OR 0·76]; 95% CI 0·68–0·84) might modulate the expression of *DIRC3*, we conducted an eQTL analysis using data from 53 tissues that were examined as part of the GTEx project.^23^ This analysis provided evidence that the effect of rs62175241 (T allele, which is protective for carpal tunnel syndrome and trigger finger, allele fraction 0·14) on *DIRC3* expression is highly tissue-specific, with positive regulation in the stomach and spleen, and negative regulation in the testes and amygdala (figure 4A). In light of evidence that *DIRC3* is able to directly activate expression of *IGFBP5*,^27^ with both genes found in the same topologically associating domain, we examined the effect of rs62175241 on *IGFBP5* expression in GTEx. This analysis again provided evidence for tissue-specific eQTL associations and showed a discordant effect of rs62175241 on *DIRC3* and *IGFBP5* expression in the spleen (figure 4B). Considering that fibroblast proliferation is a histological feature of both trigger finger and carpal tunnel syndrome,^8^ we further investigated the effect of rs62175241 on *IGFBP5* expression in fibroblasts. We re-analysed fibroblast single-cell eQTL data from 79 donors.^24^ Four of six fibroblast subtypes showed a significant positive association between the protective T allele of rs62175241 and *IGFBP5* expression (appendix p 10), with the strongest association seen in *HOXC6*+ cluster (figure 4C). LncRNAs inherently have markedly lower abundance than mRNAs; consistent with this and the shallow depth of sequencing in single cell RNA sequencing data, *DIRC3* was detected in fewer than 1% of cells, precluding further analysis.

Next, we analysed the association of rs62175241 on *DIRC3* and *IGFBP5* expression in diseased tenosynovium samples from patients with carpal tunnel syndrome (n=77). We confirmed that the protective T allele was associated with significantly increased *IGFBP5* expression (p=0·033, allele fraction 0·13; figure 4D). *DIRC3* expression levels were again low and did not show allele-specific differential expression (p=0·086, figure 4E). Because *IGFBP5* is a secreted protein, we investigated whether this variant might alter plasma concentration. We analysed a publicly available plasma proteomic GWAS dataset that was obtained from the SomaLogic platform,^28^ and found that this variant (tagged by rs10203066, A allele, *r*^2^=0·99) was associated with a non-significant increased plasma *IGFBP5* (beta=0·017; p=0·11).

As *IGFBP5* is known to directly antagonise IGF-1 signalling,^29–31^ with evidence that exogenous growth hormone treatment can cause carpal tunnel syndrome,^29^ we hypothesised that higher IGF-1 plasma levels would be associated with significantly increased risk of both trigger finger and carpal tunnel syndrome in UK Biobank participants. We identified significant associations between plasma concentrations of IGF-1 and trigger finger (hazard ratio [HR] per 1 SD 1·04 [95% CI 1·01–1·07]; p=0·02) and carpal tunnel syndrome (HR per 1 SD 1·04 [1·02–1·05]; p=4·23 × 10^–6^), which was concordant with another carpal tunnel syndrome-specific analysis in UK Biobank.^32^ If the protective effect of rs62175241 was mediated via antagonism of IGF-1 signalling, we hypothesised that this variant would be associated with the attenuation of growth hormone-regulated phenotypes such as height and lean body mass. To investigate this hypothesis, we extracted growth phenotype summary statistics from UK Biobank and selected traits that met phenome-wide significance (p<1 × 10^–5^). This process identified 22 growth-related traits that were significantly associated with rs62175241 (appendix p 11), all of which had a negative beta, including standing height (p=3·57 × 10^–18^), weight (p=8·28 × 10^–6^), forced vital capacity (p=1·14 × 10^–6^), and appendicular lean mass (p=4·90 × 10^–33^). Altogether, the available data suggest that IGF-1 is associated with increased risk of both trigger finger and carpal tunnel syndrome, and that the T allele of rs62175241 might act to directly attenuate IGF-1 signalling, thus explaining its protective effect for trigger finger and carpal tunnel syndrome.

## Discussion

**Figure 5:**
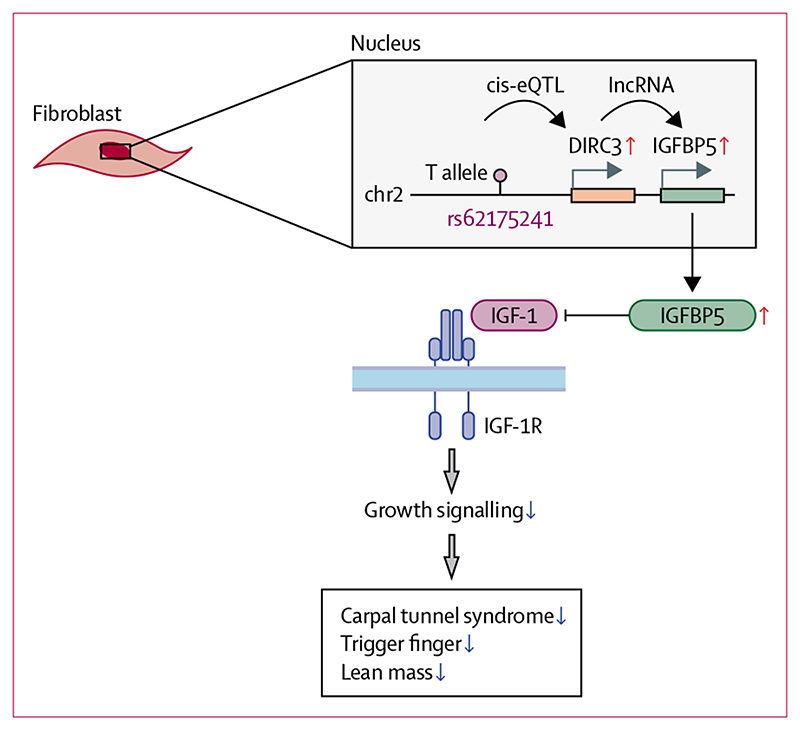
Proposed model for the role of *DIRC3* in carpal tunnel syndrome and trigger finger The disease-protective T allele of rs62175241 (*DIRC3* locus) is associated with increased expression of long non-coding RNA (lncRNA) *DIRC3* and increased RNA expression of its direct transcriptional target *IGFBP5*. IGFBP5 protein is a secreted antagonist of IGF-1 signalling that acts to suppress growth signalling. Increased plasma IGF-1 is associated with both carpal tunnel syndrome and trigger finger, suggesting that IGF-1 signalling is a driver of both conditions. We propose that the association between the T allele of rs62175241 with carpal tunnel syndrome, trigger finger, and growth phenotypes (eg, lean mass) is mediated by increased antagonist of fibroblast IGF-1 signalling, altogether directly implicating IGF-1 signalling in carpal tunnel syndrome and trigger finger pathophysiology. DIRC3=Disrupted In Renal Carcinoma 3. eQTL=expression quantitative trait. IGF-1=insulin-like growth factor-1. IGFBP5=insulin-like growth factor-binding protein-5.

This GWAS for trigger finger identified five risk loci, one of which was also identified in our previous GWAS of carpal tunnel syndrome.^10^ Hypothesising that a single genetic variant might contribute to the pathogenesis of both diseases, we fine-mapped this locus to a single putative causal SNP, rs62175241. Our single-cell eQTL analysis provided evidence to show that this variant was associated with tissue-specific modulation of the expression of *DIRC3* and its known downstream effector, *IGFBP5*. Bulk RNA sequencing analysis of surgically resected tenosynovium samples from patients with carpal tunnel syndrome showed that this protective variant was associated with enhanced expression of *IGFBP5*. Considering that IGFBP5 is an antagonist of IGF-1, we found that both trigger finger and carpal tunnel syndrome were positively associated with IGF-1 levels. These findings are important because they provide a direct biological insight into the shared pathophysiological mechanisms contributing to trigger finger and carpal tunnel syndrome (figure 5). Furthermore, our findings provide a starting point for investigating non-surgical interventions for these two common conditions.

To the best of our knowledge, the co-localised locus that mapped to the *DIRC3* gene has not been previously described in association with trigger finger. One previous GWAS^11^ was conducted to identify risk loci associated with trigger finger, finding a single genome-wide locus within *KLHL1* that did not replicate. KLHL1 is an actin-binding protein, and the authors of this study speculated that this variant might lead to fibrocartilaginous metaplasia in tenocytes.

In this GWAS, the putative causal SNP, rs62175241, is located 3731 base pairs from the canonical transcription start site of the *DIRC3* gene. The *DIRC3* locus spans 450 kilobases between the *IGFBP5* and *TNS1* genes. By mapping the chromatin structure of the *IGFBP5-DIRC3-TNS1* gene territory in human keratinocytes, Coe and colleagues^27^ showed that *DIRC3* and *IGFBP5* are located within the same topologically-associated domain. They also found two DNA looping interactions between the *DIRC3* locus and *IGFBP5* promoter. In their study, *DIRC3* levels positively correlated with *IGFBP5* in melanoma RNA sequencing samples and they discovered that *DIRC3* acts in *cis* to control expression of *IGFBP5*.

*IGFBP5* expression is altered in several fibrotic disease states. In lung tissue from patients with idiopathic pulmonary fibrosis, *IGFBP5* was upregulated and exogenous IGFBP5 stimulated extracellular matrix secretion by idiopathic pulmonary fibrosis pulmonary fibroblasts.^33,34^ Furthermore, *IGFBP5* was upregulated in skin fibroblasts from patients with systemic sclerosis.^35^ In the present study, the protective T allele at our putative causal SNP had tissue-specific effects on expression of *DIRC3* and *IGFBP5*. In both skin fibroblasts and operative tenosynovium samples from patients with carpal tunnel syndrome, the protective allele was associated with enhanced expression of *IGFBP5*. IGFBP5 is a highly conserved and multifunctional secreted protein that binds to IGF and can have complex and varying effects on IGF signalling depending on the tissue type and context. In bone, IGFBP5 inhibits IGF-1 signalling^36^ by modulating binding to the IGF-1 receptor.^30^ Similarly, in mammary tissue, IGFBP5 regulates involution by inhibiting IGF-1 signalling.^31^

Consistent with the hypothesis that overactive IGF-1 signalling is important in trigger finger and carpal tunnel syndrome, we discovered that IGF-1 plasma concentrations were positively associated with both conditions. Furthermore, despite carpal tunnel syndrome generally being associated with decreased height, the protective allele at our putative causal locus was associated with decreased height, suggesting a distinct pathophysiological mechanism.

Several other lines of evidence support the role of IGF-1 signalling in trigger finger and carpal tunnel syndrome. The prevalence of carpal tunnel syndrome^37^ and trigger finger^38^ is increased in patients with acromegaly, for whom raised IGF-1 levels are characteristic. By normalising levels of IGF-1, either through pituitary resection or somatostatin analogues, increased tendon thickness at the A1 pulley can be reversed, and symptoms of trigger finger ameliorated.^38^ In healthy patients who do not have acromegaly, administering exogenous growth hormone stimulates a rise in IGF-1, and patients can subsequently develop carpal tunnel syndrome.^29^ Exogenous growth hormone is also known to increase the risk of type 2 diabetes, which, along with type 1 diabetes, we found to be significantly enriched in patients with both carpal tunnel syndrome and trigger finger^39^ (appendix p 3). Somatostatin analogues work not only by reducing pituitary growth hormone secretion but also by stimulating IGFBP5 secretion^40^ which, in turn, inhibits IGF-1 signalling. Of note, our phenotypic analysis highlighted a significant association between trigger finger and both rheumatoid arthritis and osteoarthritis, which was even stronger in the trigger finger-carpal tunnel syndrome overlap cohort (appendix pp 3–4). IGF-1 signalling has been implicated in synovitis in both osteoarthritis^41^ and rheumatoid arthritis,^42^ which would accord with our proposed pathophysiological mechanism underlying the observed phenotypic associations.

We recognise several limitations of the present study. Firstly, our GWAS of patients with trigger finger only included patients of European ancestry. It would be helpful to further characterise the genetic architecture of trigger finger in non-European groups, who are under-represented in GWASs. Although our GWAS had a greater power (81% power) to detect a hypothetical variant with a minor allele fraction of 50% and an OR of 1·2 than a previous study (3% power),^13^ our study was still relatively underpowered, especially for low-frequency variants, meaning that other relevant risk loci might not have met our pre-defined significance threshold. Although we were able to replicate our co-localised *DIRC3* locus in patients with trigger finger and patients with carpal tunnel syndrome from the FinnGen cohort, we were unable to replicate all five trigger finger loci. This might be partly explained by the size of the replication cohort (1485 patients with trigger finger) and the use of a different case definitions in FinnGen, but might also be consistent with the so-called winner’s curse phenomenon^43^ in GWASs. Regardless, these validation data strongly support a role for the *DIRC3* locus in both trigger finger and carpal tunnel syndrome. We have identified a biologically relevant mechanism that is likely to underpin the association between our co-localised risk locus and trigger finger and carpal tunnel syndrome. However, we recognise that the association between a protective haplotype at *DIRC3*, *IGFBP5* and *IGF-1* are all correlative, and further studies are required to dissect these mechanisms and provide evidence for a causative effect.

In conclusion, we provide evidence for a phenotypic association between trigger finger and carpal tunnel syndrome in patients from the UK Biobank cohort. We identified a putative causal variant in our GWAS of trigger finger that overlaps with carpal tunnel syndrome, which possibly accounts for some of the phenotypic overlap between these two conditions. Through multimodal expression eQTL analyses, we directly linked a protective causal variant in *DIRC3* to increased expression of *IGFBP5*, an IGF-1 antagonist.^30^ Finally, we provide evidence to show a significant association between plasma IGF-1 concentrations and both trigger finger and carpal tunnel syndrome, altogether implicating IGF-1 signalling in the pathophysiology of both conditions. Collectively, our findings indicate that the protective variant at rs62175241 acts by enhancing expression of *IGFBP5*, via *DIRC3* which, in turn, inhibits IGF-1 signalling (figure 5). Further studies are required to fully characterise this pathway and delineate whether it might be a valid target for pharmacological management of trigger finger and carpal tunnel syndrome.

## Data Availability

RNA sequencing data from the Pain in Neuropathy Study (PiNS) cohort has been reported previously and is available at accession GEO108023 (https://www.ncbi.nlm.nih.gov/geo/query/acc.cgi?acc=GSE108023). The relevant raw data (count matrix and genotype calls at rs62175241) and code that are necessary to replicate analyses in the expanded Oxford-CTS RNA sequencing cohort are provided on Github (https://github.com/samkleeman1/cts_tf/). A Jupyter Notebook summarising the genotype data quality control and genome-wide association analysis implemented in Regenie is provided on Github (https://github.com/samkleeman1/cts_tf/). Single-cell fibroblast expression quantitative trait (eQTL) summary statistics and applicable source data are published alongside the original manuscript.^19^ UK Biobank data can be requested through the application process detailed at https://www.ukbiobank.ac.uk/. Summary statistics for the primary trigger finger GWAS (patients with both International Classification of Diseases[ICD]-10 and Office of Population Censuses and Surveys Classification of Surgical Operations and Procedures[OPCS]-4 codes for trigger finger) have been uploaded to the GWAS Catalog (accession code GCST90104907). Summary statistics for the sensitivity analyses described in the manuscript are available from the corresponding author on request.

## Research in context

### Evidence before this study

We did a literature review using Medline to determine the co-occurrence of trigger finger and carpal tunnel syndrome. We searched terms “trigger finger” and “carpal tunnel syndrome”, and included all studies published up to March 28, 2022. We included original research studies in all languages that quantitatively assessed co-occurrence of trigger finger and carpal tunnel syndrome, and excluded individual case reports. We identified three prospective observational studies and 24 retrospective studies. Studies report that trigger finger and carpal tunnel syndrome occur together more often than would be expected by chance.

### Added value of this study

Our study is, to the best of our knowledge, the largest genome-wide association study (GWAS) for trigger finger. We leveraged the results of this GWAS to identify a single locus (*DIRC3*) that is significantly associated with both trigger finger and carpal tunnel syndrome. We used multi-modal expression quantitative trait analysis to trace the mechanism by which *DIRC3* modifies risk for these conditions, and provide evidence to show that IGF-1 signalling has a role in disease pathophysiology.

### Implications of all the available evidence

The long-established co-occurrence of trigger finger and carpal tunnel syndrome might be at least partly explained by a shared germline predisposition, which acts to increase IGF-1 signalling in fibroblasts. Further research should determine whether this pathway might be a valid target for pharmacological management of trigger finger and carpal tunnel syndrome.
