# Supplementary material for: Shared genetic susceptibility between trigger finger and carpal tunnel syndrome: a genome-wide association study
Source: Lancet Rheumatol. 2022 Jul 21;4(8):e556–65. doi: 10.1016/S2665-9913(22)00180-1 (PMC7613465; doi:10.1016/S2665-9913(22)00180-1)
Supplement: Supplementary appendix [file mmc1.pdf]

THE LANCET
Rheumatology

Supplementary appendix

This appendix formed part of the original submission and has been peer reviewed. We post it as supplied by the authors.

Supplement to: Patel B, Kleeman SO, Neavin D, et al. Shared genetic susceptibility between trigger finger and carpal tunnel syndrome: a genome-wide association study. *Lancet Rheumatol* 2022; **4:** e556–65.

Appendix: Shared genetic susceptibility between trigger finger and carpal tunnel syndrome: a genome-wide association study

Benjamin Patel BMBCh, Sam O. Kleeman BMBCh, Drew Neavin PhD, Professor Joseph Powell PhD, Georgios Baskozos PhD, Michael Ng PhD, Waheed-Ul-Rahman Ahmed MBBS, Professor David L. Bennett PhD, Professor Annina B. Schmid PhD, Professor Dominic Furniss DM, Akira Wiberg DPhil

Page number	Nature of contents
2	Table 1: GWAS cohort definitions
3-4	Table 2: Cohort characteristics for trigger finger (TF) and carpal tunnel syndrome (CTS) cases in UK Biobank
5	Table 3: Results of association analysis for TF
6	Table 4: Sensitivity analysis showing results for each locus from primary TF GWAS using alternative case definitions
7	Table 5: Meta-analysis of UKB and FinnGen summary statistics for TF
8-9	Table 6: Finemapping of 95% credible set colocalised variants in TF-CTS genome-wide analysis
10	Table 7: Summary statistics for cis-eQTL analysis for rs62175241 on IGFBP5 in single-cell sequenced fibroblast clusters
11	Table 8: Phenome-wide association for rs62175241
12	Figure S1: CONSORT diagram summarising the selection of case and control cohorts for trigger finger genome-wide association analysis
13	Figure S2: Genomic context of rs62175241

Table 1: GWAS case definitions in UK Biobank, including the core analysis and sensitivity analyses

	**Case (n)**	**Control (n)**	**Case definition**
Core analysis	2,908	436,579	TF ICD and TF OPCS-4
Extended cohort	3,963	436,579	TF ICD or TF OPCS-4
Mixed cohort	4,512	436,579	TF ICD or TF OPCS-4 or TF self-report
TF excluding CTS	2,110	436,579	(TF ICD and TF OPCS-4), without CTS
Merged TF-CTS	16,904	436,579	(TF ICD and TF OPCS-4), or, CTS

Table 2: Cohort characteristics, for TF and CTS cases in UKB. p-value refers to Kruskal-Wallis non-parametric test (continuous variable) or chi-squared test for independence (binary variable) across all three groups.

	**Overlap TF-CTS (N = 887)**	**Carpal tunnel syndrome (N = 15,407)**	**Trigger finger (N = 2,326)**	**p-value (All)**
**Age of diagnosis**				
min	32.72	28.74	31.66	
max	77.92	78.06	77.43	
mean (sd)	57.81 ± 8.11	57.15 ± 8.92	60.09 ± 7.62	9.49×10^{-10}
**Sex**				
Male (%)	272 (31)	4,634 (30)	1,003 (43)	
Female (%)	615 (69)	10,773 (70)	1,323 (57)	2.67×10^{-35}
**BMI**				
min	17.78	13.12	15.64	
max	61	74.68	51.9	
mean (sd)	29.68 ± 5.59	29.48 ± 5.75	28.32 ± 4.83	1.71×10^{-17}
**Height**				
min	145	100	141	
max	204	200	197	
mean (sd)	164.12 ± 9.06	164.43 ± 8.72	166.76 ± 9.14	9.24×10^{-32}
**HbA1c (IFCC)**				

min	24.5	15.3	22.8	
max	125.4	132	103	
mean (sd)	40.04 ± 11.07	37.92 ± 8.44	38.75 ± 9.91	4.79×10^{-6}
**Comorbidities (%)**				
Current smoker	94 (11)	1,728 (11)	230 (10)	0.14
Previous smoker	333 (38)	5,835 (38)	959 (41)	7.00×10^{-3}
T1DM	80 (9)	524 (3)	123 (5)	5.42×10^{-19}
T2DM	191 (22)	2,476 (16)	405 (17)	5.07×10^{-5}
Hypertension	441 (50)	7,196 (47)	1,069 (46)	0.15
High cholesterol	321 (36)	5,077 (33)	888 (38)	1.26×10^{-6}
Gout	43 (5)	744 (5)	118 (5)	0.87
Osteoarthritis	463 (52)	6,749 (44)	891 (38)	2.52×10^{-12}
Hypothyroidism	1 (0)	7 (0)	0 (0)	0.36
Rheumatoid arthritis	252 (28)	3,706 (24)	486 (21)	1.96×10^{-5}

Table 3: Results of association analysis for TF. Lead SNP refers to SNP with the lowest p-value at each independent locus.

**Chromosome**	**Position (GRCh37)**	**Lead SNP**	**Effect allele**	**Non-effect allele**	**Effect allele frequency**	**Odds ratio (95% CI)**	**p-value**
2	218144954	rs10203066	A	G	0.14	0.75 (0.69–0.82)	6.73×10^{-13}
5	64783767	rs2087927	A	G	0.26	0.84 (0.79–0.89)	4.04×10^{-9}
11	65320780	rs4244811	G	A	0.33	1.17 (1.11–1.23)	3.37×10^{-9}
16	53908657	rs10521304	T	C	0.54	0.82 (0.78–0.86)	2.76×10^{-14}
16	69887707	rs3790086	C	G	0.54	0.87 (0.83–0.92)	3.61×10^{-8}

Table 4: Sensitivity analysis showing results for each locus from primary TF GWAS, using alternative case definitions, as described in Table 1. The control cohort is unchanged in each analysis. FinnGen R4 refers to our TF replication cohort, derived from the FinnGen pipeline.

					**Core analysis**		**Extended cohort**		**Mixed cohort**	
**Chromosome**	**Position (GRCh37)**	**Lead SNP**	**Effect allele**	**Non-effect allele**	**p-value**	**OR (95% CI)**	**p-value**	**OR (95% CI)**	**p-value**	**OR (95% CI)**
2	218144954	rs10203066	A	G	6.7×10^{-13}	0.75 (0.69–0.81)	1.60×10^{-11}	0.80 (0.74–0.85)	1.51×10^{-10}	0.82 (0.77–0.87)
5	64783767	rs2087927	A	G	4.04×10^{-9}	1.20 (0.78–0.88)	2.2×10^{-6}	0.88 (0.84–0.93)	9.75×10^{-7}	0.87 (0.84–0.93)
11	65320780	rs4244811	G	A	3.37×10^{-9}	1.18 (1.12–1.24)	4.64×10^{-8}	1.14 (1.09–1.19)	1.84×10^{-9}	1.14 (1.10–1.19)
16	53908657	rs10521304	T	C	2.76×10^{-14}	0.82 (0.78–0.86)	1.33×10^{-12}	0.85 (0.81–0.89)	9.83×10^{-11}	0.87 (0.84–0.91)
16	69887707	rs3790086	C	G	3.61×10^{-8}	0.87 (0.82–0.91)	2.57×10^{-7}	0.89 (0.85–0.93)	7.65×10^{-7}	0.90 (0.86–0.94)
					**TF excluding CTS**		**Merged TF-CTS**		**FinnGen R4**	
**Chromosome**	**Position (GRCh37)**	**Lead SNP**	**Effect allele**	**Non-effect allele**	**p-value**	**OR (95% CI)**	**p-value**	**OR (95% CI)**	**p-value**	**OR (95% CI)**
2	218144954	rs10203066	A	G	1.53×10^{-10}	0.74 (0.67–0.81)	1.28×10^{-24}	0.84 (0.82–0.87)	0.0055	0.88 (0.81–0.96)
5	64783767	rs2087927	A	G	1.23×10^{-4}	0.87 (0.81–0.94)	1.33×10^{-5}	0.95 (0.92–0.97)	0.6166	0.98 (0.90–1.07)
11	65320780	rs4244811	G	A	1.05×10^{-9}	1.22 (1.14–1.29)	8.49×10^{-6}	1.05 (1.03–1.08)	0.0205	0.90 (0.82–0.98)
16	53908657	rs10521304	T	C	2.18×10^{-13}	0.80 (0.75–0.84)	1.80×10^{-1}	0.98 (0.96–1.00)	0.0562	0.93 (0.86–1.00)
16	69887707	rs3790086	C	G	1.94×10^{-11}	0.81 (0.77–0.86)	1.84×10^{-3}	0.97 (0.94–0.99)	0.0009	0.88 (0.82–0.95)

Table 5: Meta-analysis of UKB and FinnGen summary statistics for TF. Direction refers to either risk-increasing (+) or risk-reducing (-) effects of Allele 1. There was directional concordance between UKB and FinnGen at all five loci.

**SNP**	**Allele 1**	**Allele 2**	**Zscore**	**p-value**	**Direction**	**HetISq[a]**	**HetChiSq[b]**	**HetDf[c]**	**HetPVal[d]**
rs10203066	A	G	-7.62	2.4×10^{-14}	--	17.3	1.21	1	0.271
rs2087927	A	G	-5.37	7.70×10^{-8}	--	83.3	5.97	1	0.0145
rs4244811	G	A	6.29	3.18×10^{-10}	++	0	0.765	1	0.382
rs10521304	T	C	-7.57	3.75×10^{-14}	--	76.5	4.25	1	0.0392
rs3790086	C	G	-6.43	1.24×10^{-10}	--	0	0.0430	1	0.835

[a]HetISq is the heterogeneity of I^2 parameter
[b]HetChiSq is the heterogeneity of Chi^2 parameter
[c]HetDf is the heterogeneity of degrees of freedom
[d]HetPVal is the heterogeneity of P value

Table 6: Fine-mapping of 95% credible set colocalised variants in TF-CTS genome-wide analysis.

Chr	Position	A0	A1	SNP	A1 frequency	INFO score	Beta	SE	p-value	Consequence	Gene
2	218134988	T	C	rs62175241	0.856	0.998	0.285	0.041	1.01×10^{-12}	TF_binding_ site_variant	-
2	218120660	A	G	rs12622529	0.856	0.994	0.283	0.041	1.50×10^{-12}	regulatory_ region_variant	-
2	218121776	A	G	rs4674138	0.856	0.995	0.280	0.041	2.62×10^{-12}	intergenic_ variant	-
2	218121903	A	G	rs56733382	0.856	0.995	0.281	0.041	2.65×10^{-12}	intergenic_ variant	-
2	218126236	C	T	rs10490757	0.856	0.998	0.279	0.041	2.99×10^{-12}	regulatory_ region_variant	-
2	218127033	T	C	rs62175239	0.856	0.998	0.286	0.041	9.38×10^{-13}	intergenic_ variant	-
2	218129547	G	A	rs4674139	0.856	0.998	0.284	0.041	1.29×10^{-12}	intergenic_ variant	-
2	218134786	T	G	rs62175240	0.856	0.998	0.285	0.041	1.0×10^{-12}	intergenic_ variant	-
2	218138484	C	T	rs11695415	0.856	0.998	0.280	0.041	2.19×10^{-12}	intergenic_ variant	-
2	218138857	C	G	rs60279308	0.856	0.998	0.282	0.041	1.75×10^{-12}	intergenic_ variant	-
2	218139894	G	C	rs10490758	0.855	0.997	0.282	0.041	1.61×10^{-12}	intergenic_ variant	-
2	218140435	C	T	rs11676136	0.856	0.998	0.280	0.041	2.13×10^{-12}	intergenic_ variant	-
2	218142424	C	A	rs16857158	0.856	0.998	0.280	0.041	2.25×10^{-12}	regulatory_ region_variant	-
2	218143731	T	C	rs2113825	0.856	0.998	0.280	0.041	2.15×10^{-12}	upstream_ gene_variant	*AC009492.1*
2	218144954	A	G	rs10203066	0.856	1.000	0.288	0.041	6.73×10^{-13}	regulatory_ region_variant	-

2	218145707	A	G	rs10221930	0.856	0.999	0.281	0.041	2.09×10^{-12}	downstream_ gene_variant	*DIRC3*
2	218146108	C	G	rs10221933	0.857	0.999	0.279	0.041	3.17×10^{-12}	downstream_ gene_variant	*DIRC3*
2	218146354	G	T	rs57632285	0.856	0.998	0.280	0.041	2.4×10^{-12}	downstream_ gene_variant	*DIRC3*
2	218146382	G	A	rs57794967	0.856	0.998	0.280	0.041	2.46×10^{-12}	downstream_ gene_variant	*DIRC3*
2	218147429	G	A	rs11683981	0.856	0.998	0.281	0.041	2.26×10^{-12}	regulatory_ region_variant	-
2	218148030	G	C	rs6713558	0.856	0.998	0.279	0.041	2.91×10^{-12}	downstream_ gene_variant	*DIRC3*

Table 7: Summary statistics for cis-expression quantitative trait loci (eQTL) analysis for our fine-mapped SNP, rs62175241, on Insulin Like Growth Factor Binding Protein-5 (IGFBP5) in single-cell sequenced fibroblast clusters from 79 donors.

**Cell cluster**	**SNP**	**Beta**	**t-stat**	**p-value**
HOXC6+	2:218134988_T	0.927	4.607	1.62×10^{-5}
ATF1+	2:218134988_T	0.835	4.055	1.20×10^{-4}
TEAD2+	2:218134988_T	0.654	3.018	3.46×10^{-3}
SIX5+	2:218134988_T	0.632	2.948	4.24×10^{-3}
KLF10+	2:218134988_T	0.517	2.257	2.69×10^{-3}
RXRB+	2:218134988_T	0.451	1.606	0.113

Table 8: Phenome-wide association for the fine-mapped SNP, rs62175241, with data derived from OpenTargets Genetics Portal. Data ordered by increasing p-value.

Study ID	Trait	p-value	Beta	N Overall
GCST90000025	Appendicular lean mass	4.90×10^{-33}	-0.032	450243
NEALE2_50_raw	Standing height	3.57×10^{-18}	-0.185	360388
NEALE2_23129_raw	Trunk fat-free mass	1.40×10^{-16}	-0.090	354530
NEALE2_23130_raw	Trunk predicted mass	1.96×10^{-16}	-0.086	354494
NEALE2_23101_raw	Whole body fat-free mass	3.04×10^{-15}	-0.167	354808
NEALE2_23102_raw	Whole body water mass	3.39×10^{-15}	-0.123	354834
NEALE2_23122_raw	Arm predicted mass (right)	6.55×10^{-13}	-0.010	354726
NEALE2_23105_raw	Basal metabolic rate	6.68×10^{-13}	-20.402	354825
NEALE2_23113_raw	Leg fat-free mass (right)	6.92×10^{-13}	-0.029	354798
NEALE2_23126_raw	Arm predicted mass (left)	7.15×10^{-13}	-0.010	354653
NEALE2_23114_raw	Leg predicted mass (right)	9.27×10^{-13}	-0.027	354798
NEALE2_23125_raw	Arm fat-free mass (left)	1.05×10^{-12}	-0.011	354668
NEALE2_23121_raw	Arm fat-free mass (right)	1.11×10^{-12}	-0.010	354732
NEALE2_20015_raw	Sitting height	1.83×10^{-11}	-0.082	360066
NEALE2_23118_raw	Leg predicted mass (left)	1.38×10^{-10}	-0.024	354766
NEALE2_23117_raw	Leg fat-free mass (left)	1.85×10^{-10}	-0.026	354771
NEALE2_46_raw	Hand grip strength (left)	3.01×10^{-9}	-0.151	359704
NEALE2_1697	Comparative height size at age 10	6.10×10^{-9}	-0.013	355331
NEALE2_47_raw	Hand grip strength (right)	2.27×10^{-7}	-0.132	359729
NEALE2_3062_raw	Forced vital capacity (fvc)	1.14×10^{-6}	-0.013	329404
NEALE2_3063_raw	Forced expiratory volume in 1-second (fev1)	3.67×10^{-6}	-0.009	329404
NEALE2_21002_raw	Weight	8.28×10^{-6}	-0.212	360116

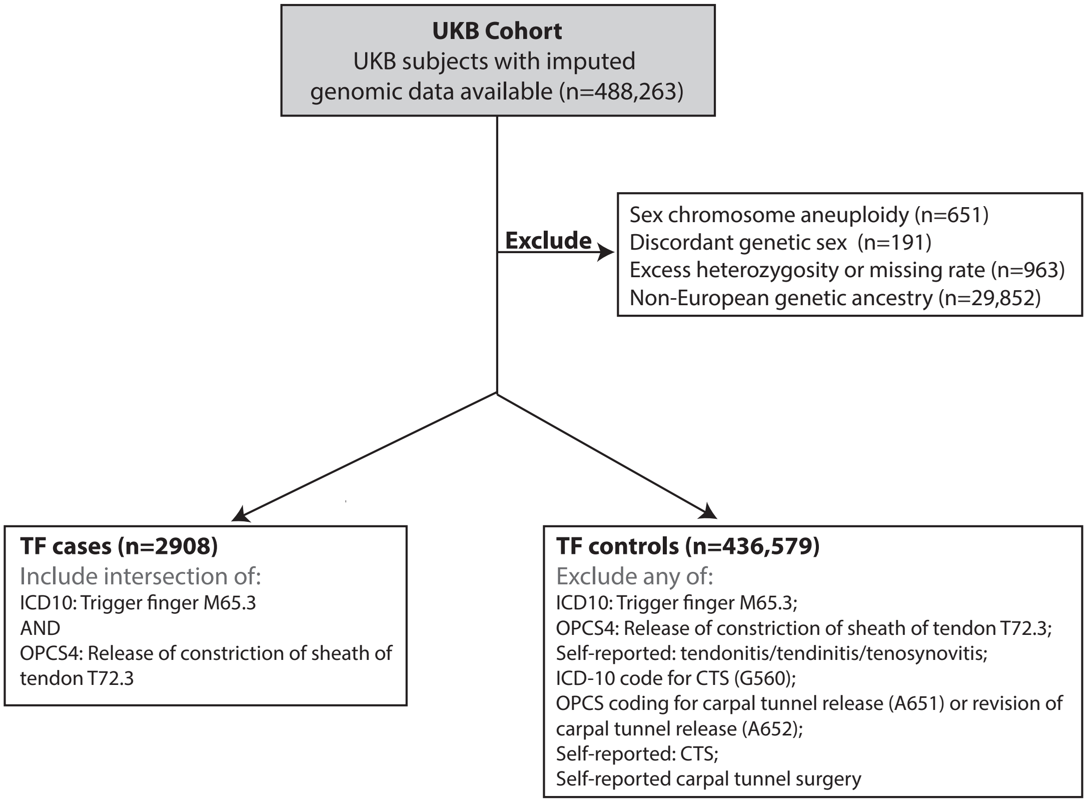

Figure S1: CONSORT diagram summarizing the selection of case (n=2908) and control (n=436,579) cohorts for trigger finger (TF) genome-wide association analysis in European subjects from UKB cohort.

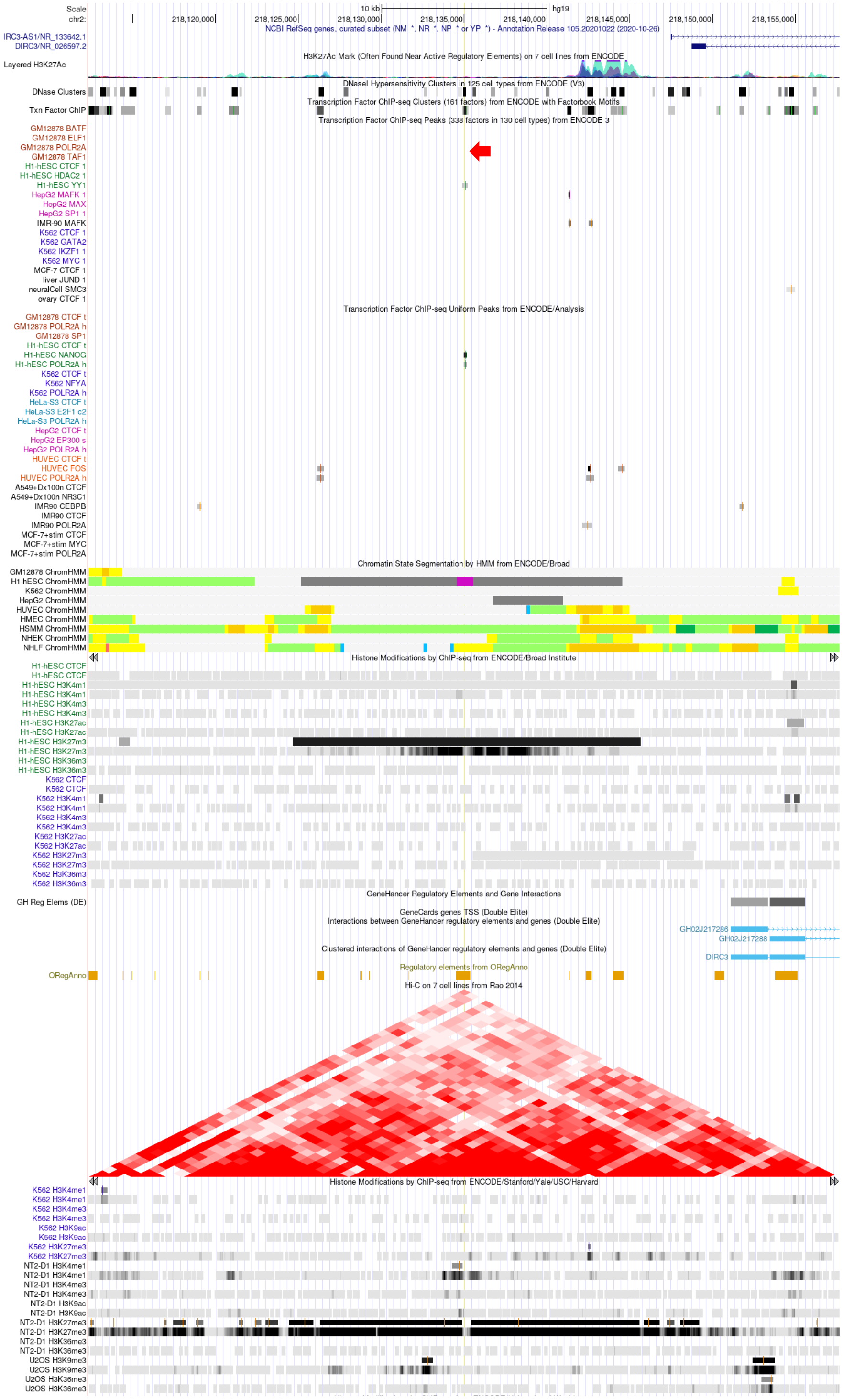

Figure S2: Genomic context of rs62175241 (marked with arrow), annotated with DIRC3 transcript location, functional genomics annotations including transcription factor and histone modification ChIP-seq, DNase-accessible sites, predicted regulatory elements and Hi-C data indicating chromosome spatial organization. Credit UCSC Genome Browser (https://genome.ucsc.edu/index.html).
